# Referees

**DOI:** 10.1002/rmb2.12492

**Published:** 2023-01-23

The publication of invaluable papers in the Reproductive Medicine and Biology depends on the prompt, careful review of submitted manuscripts. We would like to thank the Editorial Board members, the Editorial Advisory Board members and the following experts for reviewing manuscripts from November 1, 2021 to October 31, 2022.

Hidenori Akutsu

Ahmed T Alahmar

kristian Almstrup

Yoshihiko Araki

Yoshiyuki Asai

Atsushi Azumaguchi

Tsuyoshi Baba

Fumihisa Chishima

Maryse Delehedde

Ludovic Dumont

Toshiaki Endo

Masakatsu Fujinoki

Satoko Fujioka

Maki Fukami

Rie Fukuhara

Atsushi Fukui

Hiroaki Funahashi

Tatsuro Furui

Sun‐Wei Guo

Jun Hagiuda

Kenshiro Hara

Akiko Harada

Miyuki Harada

Katherine E Hartmann

Hisataka Hasegawa

Shu Hashimoto

Satoshi Hayakawa

Yuji Hirao

Yasushi Hirota

Akihito Horie

Kuei‐Yang Hsiao

Kentaro Ichioka

Takuhiko Ichiyama

Kazuhiko Imakawa

Osamu Ishihara

Hiroshi Ishikawa

Tomomoto Ishikawa

Nobuhiko Itami

Junya Ito

Masahiro Itoh

Fumiaki Itoi

Takeshi Iwasa

Shoichiro Iwatsuki

Gentaro Izumi

Chiaki Izumiya

Seung Chik Jwa

Daisuke Kadogami

Takeshi Kajihara

Hiroaki Kajiyama

Yasuhiko kamada

Haruhiko Kanasaki

Masahiro Kaneda

Keiichi Kato

Satoshi Kawachiya

Kazuhiro Kawamura

Natsuko Kawano

Yasushi Kawano

Khaleque Khan

Fuminori Kimura

Hiroshi Kishi

Michio Kitajima

Hideyuki Kobayashi

Ko Kobayashi

Yoshitomo Kobori

Kaori Koga

Shouhei Komemoto

Shinnosuke Komiya

Fumikazu Kotsuji

Takeshi Kuramoto

Keiji Kuroda

Shin‐no‐suke Kuroda

Satoshi Kurosaka

Yoshimitsu Kuwabara

Tomoichiro Kuwazuru

Hirohisa Kyogoku

Koichi Kyono

Kate L. Loveland

Eri Maeda

Ryo Maekawa

Hirotaka Masuda

Hidehiko Matsubayashi

Hiromichi Matsumoto

Shinya Matsuzaki

Ewa Rajpert‐De Meyts

Yasuyuki Mio

Hidehiko Miyake

Akio Miyamoto

Haruhiko Miyata

Shigetoshi Mizumoto

Satoshi Mizuno

Taisuke Mori

Tetsunori Mukaida

Srabani Mukherjee

Yumi Nagata

Koji Nakagawa

Tomoko Nakamura

Tatsuya Nakano

Yoshiharu Nakaoka

Akitoshi Nakashima

Yasuo Nambo

Kaei Nasu

Takuji Nishihara

Taichi Noda

Junko Noguchi

Masanori Ochi

Satoshi Ohkura

Hidetaka Okada

Osamu Okitsu

Masanori Ono

Aki Oride

Makoto Orisaka

Satoko Osuka

Kuniaki Ota

Nobuaki Ozawa

Davood Rostamzadeh

Osamu Samura

Attilio Di Spiezio Sardo

Shun Sato

Takeshi Sato

Takahiko Shimizu

Masahide Shiotani

Hiromitsu Shirasawa

Tetsuji Soda

Yodo Sugishita

Masahito Tachibana

Seido Takae

Toshifumi Takahashi

Yasushi Takai

Masashi Takamura

Kumiko Takeda

Teppei Takeshima

Isao Tamura

Kazuhiro Tamura

Fuminori Taniguchi

Kenji Tanimura

Satoshi Tanimura

Hidetaka Tasaki

Fumiko Tawara

Masakazu Terauchi

Hiroyuki Tomari

Kazuhisa Tomita

Isao Tsuji

Takashi Umehara

Takafumi Utsunomiya

Osamu Wada‐Hiraike

Tomohiko Wakayama

Seiji Watanabe

Mitsutoshi Yamada

Yuri Yamamoto

Kazumitsu Yamasaki

Yasuhisa Yamashita

Marc Yeste

Keiichiro Yogo

Manabu Yoshida

Yushi Yoshimasa

Osamu Yoshino

Yasushi Yumura

